# A rare case of vision loss caused by leptomeningeal metastasis of lung adenocarcinoma: a case report and literature review

**DOI:** 10.3389/fonc.2025.1484383

**Published:** 2025-02-10

**Authors:** Zhihua Li, Jian Chu, Wennan Shen, Junnan Chen, Yuemei Dong, Manze Zhang, Nan Zhao, Wei Zhao, Haoran Zha, Ning Wang, Yalin Han, Zhaoxia Li

**Affiliations:** Department of Oncology, Chinese People's Liberation Army Rocket Force Characteristic Medical Center, Beijing, China

**Keywords:** leptomeningeal metastasis, BRAF non-V600E mutation, NSCLC, trametinib, vision loss, case report

## Abstract

Leptomeningeal metastasis (LM) is a fatal complication with increasing incidence in patients with non-small cell lung cancer. Herein, we present the case of a patient who presented with complete vision loss due to LM and achieved a survival benefit from treatment with trametinib. The treatment was prescribed based on the detection of a BRAF non-V600E mutation in the cerebrospinal fluid (CSF). We reviewed the literature and evaluated survival benefits in patients with LM harboring BRAF non-V600E mutations treated with CSF chemotherapy and mitogen-activated extracellular signal-regulated kinase inhibitors.

## 1 Introduction

Patients with non-small cell lung cancer (NSCLC) have a high incidence of central nervous system metastases, with approximately 3–10% developing leptomeningeal metastasis (LM) over the course of the disease (1, 2). Notably, 20% of these cases occur at the time of initial lung cancer diagnosis (3). Additionally, LM is generally considered the end stage of tumor progression and remains difficult to treat, despite modern strategies that have extended patients’ overall survival (OS) from 1–3 to 3–11 months (4). Diagnosing LM remains challenging owing to its heterogeneous presenting signs and symptoms. It requires a comprehensive neurological examination, magnetic resonance imaging (MRI) of the brain and spine, and cerebrospinal fluid (CSF) analysis. Gadolinium-enhanced MRI of the brain and spine is the most effective imaging technique for evaluating LM. MRI findings indicative of LM include pathological enhancement of the leptomeninges in the brain, cranial nerves, and spinal cord, appearing as nodular, linear, arched, focal, or diffuse intensifications (5). While positive CSF cytology remains the gold standard for diagnosing LM, repeated CSF sampling through lumbar puncture is often necessary. The sensitivity of the initial lumbar puncture has been reported to be as low as 50%, which could increase to 75–85% with a second CSF analysis (6). Prior to the advent of tyrosine kinase inhibitors (TKIs), LM treatment showed limited improvement in OS and involved intrathecal chemotherapy (ITC), whole-brain radiation therapy (WBRT), and systemic chemotherapy (7). Patients harboring oncogene driver mutations are more likely to develop meningeal metastases, especially those carrying epidermal growth factor receptor (EGFR) mutations (9.4% vs. 1.7%; p < 0.001) (8). In recent years, EGFR-TKIs targeting EGFR-activating mutations have been approved, demonstrating improved ability to cross the blood-brain barrier. These agents offer promising treatment options for patients with NSCLC and LM (9, 10). However, further exploration of treatment options is necessary for patients without EGFR mutations. Moreover, previous studies have demonstrated significant genomic divergence between primary tumors and intracranial metastases (11). Consequently, providing individualized therapy for patients with LM remains an exceptionally challenging task for physicians.

Vision loss caused by intracranial hypertension is rarely reported in patients with NSCLC (12, 13). Several studies have demonstrated that genetic results obtained from the CSF can be utilized to direct the course of treatment (14, 15). However, there have been few reports of targeted therapies for BRAF non-V600E mutations based on CSF genetic testing in NSCLC (16). This case report patient presented with a headache as the initial symptom of LM resulting from lung adenocarcinoma and was treated with the MET inhibitor, trametinib. This study aimed to provide insights and valuable information for the management of patients with NSCLC and LM.

## 2 Case description

**Figure 1:**
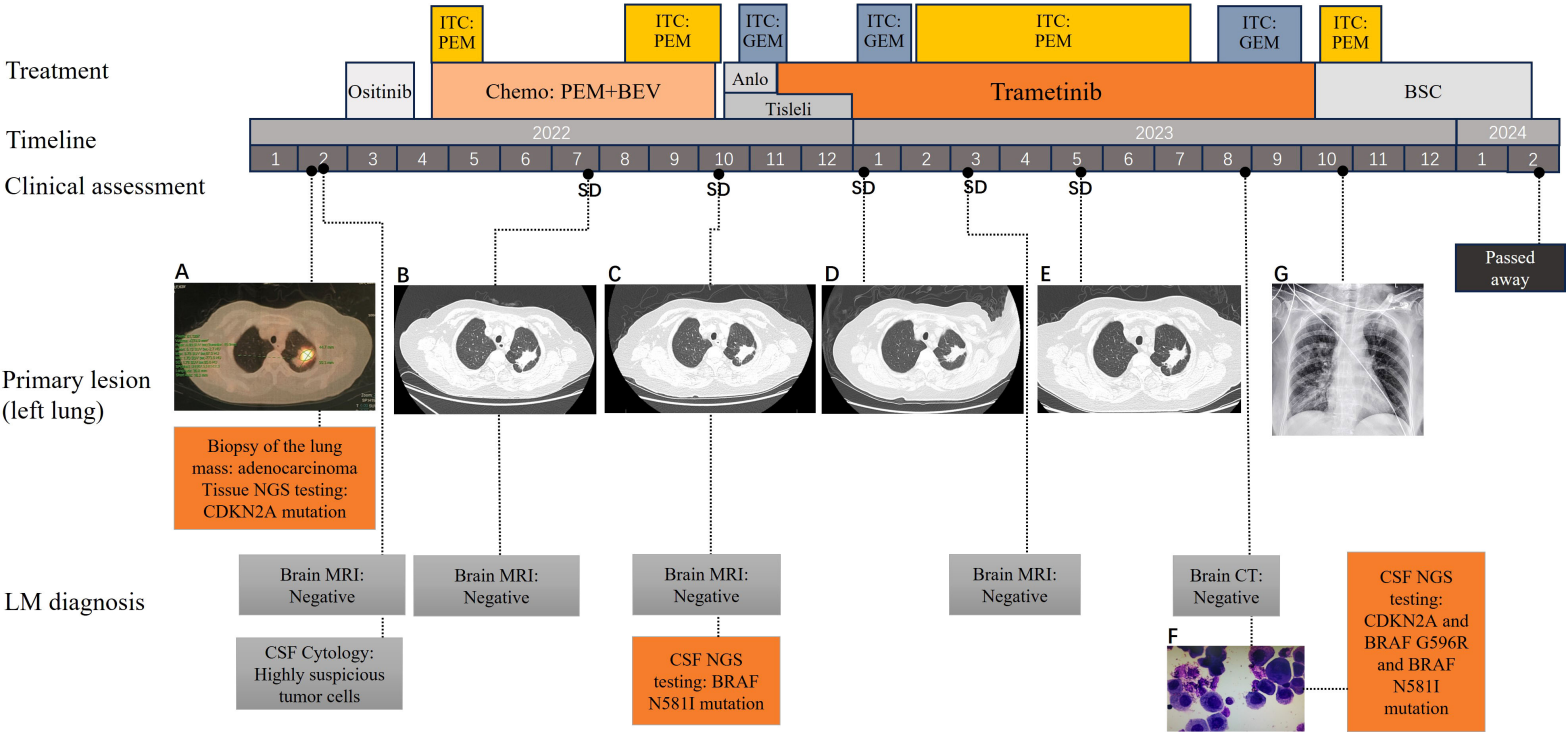
Treatment history, CT and MRI scans, and gene mutations of the patient at different clinical time points. **(A)** PET-CT scans showing the primary tumor mass (biopsy site) in the left lung at the time of diagnosis; **(B–E)** chest CT revealing SD following treatments; **(F)** Malignant tumor cells were found in the CSF; **(G)** Chest DR showing no significant progression in the lungs; CT scans were not conducted owing to the patient’s frailty. CT, computed tomography; PET-CT, Positron emission tomography-CT; SD, stable disease; CSF, cerebrospinal fluid; DR, Digital Radiography; SD, stable disease.

A 63-year-old woman with no history of smoking visited the neurology clinic of Xuanwu Hospital of Capital Medical University in February 2022 with the main complaints of progressive headache and vomiting for 3 months, which had worsened over a month. The patient had visited other hospitals several times. Her medical history indicated hypertension for 10 years, which was controlled with oral medication, with no family history of tumors. Brain computed tomography (CT) did not reveal any significant abnormalities, while contrast-enhanced brain MRI showed bilateral frontal-parietal subcortical punctate ischemic foci. The symptoms did not improve significantly after symptomatic treatment. Lumbar puncture showed increased intracranial pressure (>330 mmH_2_O) with highly suspicious tumor cells in the CSF. Further F^18^-Fluorodeoxyglucose positron emission tomography CT revealed a solid mass in the upper lobe of the left lung, which was suggestive of malignancy (**Figure 1A**). The patient was transferred to the Peking University Cancer Hospital, where a biopsy of the lung mass revealed adenocarcinoma. Immunohistochemistry was negative for anaplastic lymphoma kinase (ALK), ROS proto-oncogene 1, and programmed death-ligand 1 (PD-L1). Biopsy specimens were analyzed using next-generation sequencing to identify possible targetable molecular alterations. The findings revealed CDKN2A nonsense mutation variant allele fraction (VAF) 17.08% and ARID5B, NF1, and TGFBR1 mutations (VAF ranging from 7.25–17.53%). The patient declined chemotherapy and, since the end of February 2022, began taking ositinib orally (160 mg, 1/day) on her own. By April 2022, her headache and vomiting had worsened, and a repeat chest CT showed no changes compared to the previous scan.

The patient visited our department for further treatment, and we confirmed the diagnosis of lung adenocarcinoma with LM. According to the Eighth Edition of the TNM Classification for Lung Cancer, the patient was classified as having stage CT3NxM1a lung cancer. To promptly alleviate the patient’s cranial hypertension symptoms, we performed a lumbar puncture on April 24, 2022. CSF pressure was measured to be >330 mmH_2_O. Thirty milliliters of CSF was released, and ITC was administered, consisting of dexamethasone 5 mg in combination with pemetrexed 50 mg (day 1, day 10). The patient’s headache and vomiting symptoms improved significantly, and no malignant tumor cells were detected in the CSF. The concomitant systemic therapy consisted of pemetrexed combined with bevacizumab as first-line treatment and maintenance therapy, with the efficacy evaluated as stable disease. The patient’s headache and vomiting worsened by the end of cycle 6. Furthermore, the ITC regimen was administered multiple times. During follow-up, the patient’s cephalic symptoms and lung lesions remained stable. Cranial MRI scans showed no clear manifestations of brain parenchymal or meningeal metastases, and CSF analysis revealed no malignant tumor cells.

In October 2022, the patient presented with worsening headache, vomiting, and transient loss of consciousness. The ITC regimen was switched to gemcitabine (20 mg, 1/week) owing to poor response to pemetrexed, resulting in relief of the patient’s headache and vomiting symptoms. Simultaneously, intravenous PD-1 inhibitor tislelizumab (200 mg, 1/21 days) combined with an oral anlotinib (8 mg, 1/day) regimen was used as second-line systemic therapy, but anilotinib was discontinued after one cycle owing to uncontrolled hypertension. CSF genetic testing using second-generation sequencing technology revealed potentially clinically significant class II mutations, including the serine/threonine-protein kinase B-raf (BRAF) N581I missense mutation (abundance 13.93%) and the CDKN2A mutation (abundance 39.21%). Based on the patient’s physical status and genetic testing results of the CSF, she was started on the oral mitogen-activated extracellular signal-regulated kinase inhibitor trametinib (2 mg, once daily) as third-line systemic therapy on November 16, 2022. We continued administering ITC using gemcitabine at a dose of 20 mg every 1–2 weeks.

In January 2023, the patient’s headaches and vomiting worsened; her blood pressure increased significantly, and the CSF pressure exceeded 330 mmH_2_O. Consequently, we changed the ITC regimen to pemetrexed 30 mg every 1–2 weeks, which significantly improved the patient’s symptoms. In April 2023, the patient experienced symmetrical decline in vision in both eyes. Initially, the vision was blurred; by May 2023, the patient was essentially blind in both eyes. Funduscopic examination by an ophthalmologist revealed severe bilateral optic disc edema, optic neuropathy, and retinal vascular occlusion associated with high intracranial pressure. In June 2023, the patient underwent lateral ventricular Ommaya capsule implantation. The CSF pressure was measured through the Ommaya capsule and found to be 320 mm H2O. Subsequently, the patient was administered ITC (pemetrexed 30 mg every 1–2 weeks) through an Ommaya capsule. In August 2023, the patient’s neurological symptoms worsened, including disorientation, confused speech, and hallucinations. Additionally, a significant number of malignant tumor cells was found in the CSF (**Figure 1F**). Further genetic testing of the CSF revealed CDKN2A nonsense mutation (VAF 66.74%), BRAF G596R mutation (VAF 20.58%), BRAF N581I (VAF 1.83%), BRAF K483E (VAF 1.14%), BRAF G466R (VAF 0.75%), and RET fusion mutation (VAF 0.25%). However, cranial MRI did not reveal any clear signs of tumor metastasis. Considering that the efficacy of ITC had deteriorated again, pemetrexed was replaced with gemcitabine. The patient’s neurological symptoms and headache were significantly relieved, except for visual status.

This regimen was continued until October 2023, when it was terminated owing to the development of pulmonary infection and bacteremia, as well as deterioration in physical status. The patient died in February 2024. The patient survived for 24 months after experiencing LM. The patient continued trametinib for approximately 10 months, during which the disease remained stable on regular follow-up lung CT and cranial MRI, although the expected disease remission did not occur. The treatment timeline is shown in **Figure 1**.

## 3 Discussion

In this study, we reported a rare case of vision loss caused by intracranial hypertension owing to LM resulting from lung adenocarcinoma. Based on the detection of BRAF gene mutations in the patient’s CSF, targeted therapy with mitogen-activated extracellular signal-regulated kinase inhibitors was administered, resulting in significant survival benefits.

LM is a devastating complication of systemic cancer and typically manifests in the advanced stages of malignant tumors rather than in the early stages (17). Cases of lung adenocarcinoma presenting with headache as an initial symptom due to LM are relatively rare. A *post-hoc* analysis including 1148 patients from four randomized clinical trials showed that median time to development of LM is 14.92 months (interquartile range: 7.7–21.84) (18). In addition to clinical manifestations, an accurate diagnosis of LM relies on complementary CSF cytology and neuroimaging (19). Reportedly, 20–30% of patients with confirmed LM have a false-negative MRI (20). A positive CSF cytology is found on the initial lumbar puncture in 50% of patients with LM and in approximately 75–85% of patients who undergo two lumbar punctures (6, 21). The complementary use of these two diagnostic tools could facilitate the detection of LM in clinical practice. LM is characterized by a combination of cerebral, cranial nerve, and spinal signs and symptoms that develop either simultaneously or sequentially. These symptoms can be difficult to distinguish from those caused by malignancies at other sites or the adverse effects of cancer treatment (22). Idiopathic intracranial hypertension, another disease that affects the central nervous system, can lead to vision loss. It is estimated that approximately 10% of patients with idiopathic intracranial hypertension will eventually experience blindness in both eyes because of chronic optic disc edema (23). LM should be considered in any patient experiencing acute visual loss, diplopia, visual field defects, or optic neuropathy without an intraocular cause, particularly if accompanied by multifocal neurological deficits or a history of cancer (24). Ophthalmological examination of our patient revealed severe edema of the fundus, bilateral optic papillae, bilateral optic neuropathy, and retinal vascular occlusion in both eyes, which was considered the direct cause of blindness.

The patient attempted self-treatment with oral ositinib, on her own initiative, without any benefit. This outcome was expected because the patient did not harbor an EGFR mutation. TKIs offer improved remission and survival benefits for patients with NSCLC and LM who harbor EGFR mutations and ALK rearrangements (25, 26). A retrospective analysis showed that EGFR TKI therapy is an independent predictor of prolonged survival in patients with NSCLC carrying EGFR mutations. Patients who receive EGFR TKI therapy after LM diagnosis have longer OS than those who do not (median 9.5 versus 1.7 months) (27). Treatment options for patients without actionable mutations include systemic chemotherapy, intrathecal injections, whole brain radiotherapy (WBRT), and immune checkpoint inhibitors (ICIs) therapy. Systemic chemotherapy is the preferred treatment of choice for patients with LM resulting from NSCLC with no actionable mutations, as it has been reported to be an independent predictor of survival (20). In an analysis of 50 patients diagnosed with NSCLC-LM, Park et al. reported that patients who receive systemic chemotherapy have longer survival than those who do not (11.5 vs. 2.1 months) (28). ICIs therapy, although associated with lower response rates, may achieve better remission times in some patients with NSCLC-LM (29). PD-1/PD-L1 antibodies (e.g., nivolumab and pembrolizumab) cannot easily penetrate the blood–brain barrier because of their high molecular weight (>140000 Da) and hence act through systemic activation of immune cells. Further screening for biomarkers in populations preferring ICIs is necessary.

Although ITC may achieve higher CSF drug concentrations and is a potentially effective treatment for patients with LM from NSCLC, the optimal agent, dosing, and schedule have yet to be defined (30). Currently, there is no high-level evidence of an improvement in OS or progression-free survival (PFS) with ITC use (31). Pemetrexed and gemcitabine were used as ITC drugs in this study. The efficacy of intrathecal pemetrexed in patients with LM from NSCLC has been confirmed in a phase II clinical study (32). Although no clinical trials have confirmed the efficacy of intrathecal gemcitabine, it has been shown to be an effective systemic treatment for NSCLC (33). Considering the rapid metabolism of chemotherapeutic agents in CSF and the potential for drug resistance, ITC was administered in low- and high-frequency modes. Additionally, the two chemotherapeutic drugs were alternated based on the patient’s symptoms to delay drug resistance and minimize toxic side effects. To rapidly reduce intracranial pressure and alleviate the patient’s cranial symptoms, we slowly released 30 ml of CSF before administering intrathecal injections of chemotherapy drugs. The patient benefited from ITC in terms of the rapid reduction in intracranial pressure and control of local tumor growth. WBRT was not administered to the patient because of her decision and the controversial efficacy of WBRT in treating LM (7, 34).

The patient underwent both tissue- and CSF-based genetic testing for the identification of targetable somatic mutations. Different genetic mutations were found in the CSF compared to the tissue. Several studies have demonstrated more comprehensive profiles of driver and resistance genes in the CSF than in the plasma or primary lesions in LM diagnosis (35, 36). Circulating cell-free DNA (cfDNA) in the CSF cannot fully circulate in the blood owing to the blood-CSF barrier, resulting in an extremely small amount of cfDNA from the CNS being released into the plasma. Simultaneously, ctDNA from the primary tumor or other extracranial metastases can partially interfere with cfDNA in the CSF. Therefore, gene expression profiles in the plasma may not fully reflect the molecular profile of LM (37, 38).

BRAF mutations occur in approximately 2–4% of patients with lung adenocarcinoma and are mutually exclusive of EGFR, KRAS, and EML4–ALK (39, 40). Among patients with NSCLC harboring BRAF mutations, ~50% have the BRAF V600E mutation, which activates BRAF in its monomeric state and makes them sensitive to BRAF mutant-specific inhibitors. In a multicenter, single arm, non-randomized phase II study, dabrafenib was administered in combination with trametinib in patients previously treated with BRAF V600E-mutant NSCLC. This resulted in an ORR of 63.2% and a DCR of 79%. The median OS was 9.7 months, and 65% of the patients achieved PFS of >6 months (41). Therefore, multiple guidelines recommend the combination of dabrafenib and trametinib as first-line or subsequent therapy for patients with metastatic NSCLC harboring the BRAF V600E mutation.

BRAF non-V600E mutations occur in 1–2% of patients with NSCLC and are more likely to be observed in smokers, as compared V600E mutations. Because of the rarity of these mutations, their associated clinical features and prognostic significance have not been thoroughly described (42). Johnson et al. (43) analyzed the efficacy of trametinib in patients with BRAF non-V600E mutant tumors, including nine patients with lung adenocarcinoma. There were no complete responses; one patient (3%) had a confirmed partial response (a patient with breast ductal adenocarcinoma and a *BRAF* G469E mutation), while 10 patients had stable disease as their best response (clinical benefit rate 34%). The median PFS was 1.8 months, and the median OS was 5.7 months. Although the final conclusions of the study indicated that trametinib does not demonstrate significant clinical activity in patients with non-V600 BRAF mutations, a patient with NSCLC carrying the BRAF G469A mutation achieved a PFS of 20.4 months. Another preclinical study targeting the BRAF non-V600E mutation was the newly discovered pan-RAF drug LY3009120. This study concluded that LY3009120 is effective in inhibiting NSCLC cells harboring the BRAF non-V600E mutation and is a potent therapeutic agent for patients with BRAF non-V600E mutant NSCLC (44). These results are particularly noteworthy because of the limited data and the clear unmet need for effective targeted therapy in patients with BRAF-mutant NSCLC.

In summary, there are no reports or relevant clinical data analyses regarding patients with LM harboring BRAF non-V600E mutations being treated with trametinib. Exploring the treatment strategies for patients with targetable gene mutations is critical, particularly for those with LM. CSF genetic testing plays an important role in guiding individualized treatment of patients with CNS metastases, as appropriate targeted treatments can be selected based on testing results. Trametinib may provide a new therapeutic option for patients with LM and lung adenocarcinoma carrying BRAF non-V600E mutations. However, large prospective studies are warranted to further evaluate its efficacy.

## Data Availability

The original contributions presented in the study are included in the article/supplementary material. Further inquiries can be directed to the corresponding authors.

## References

[1] Alexander M Lin E Cheng H . Leptomeningeal metastases in non-small cell lung cancer: optimal systemic management in NSCLC with and without driver mutations. Curr Treat Opt Oncol. (2020) 21:72. doi: 10.1007/s11864-020-00759-3 32725549

[2] Cheng H Perez-Soler R . Leptomeningeal metastases in non-small-cell lung cancer. Lancet Oncol. (2018) 19:e43–55. doi: 10.1016/S1470-2045(17)30689-7 29304362

[3] Eichler AF Loeffler JS . Multidisciplinary management of brain metastases. Oncol. (2007) 12:884–98. doi: 10.1634/theoncologist.12-7-884 17673619

[4] Yin K Li Y-S Zheng M-M Jiang B-Y Li W-F Yang J-J . A molecular graded prognostic assessment (molGPA) model specific for estimating survival in lung cancer patients with leptomeningeal metastases. Lung Cancer Amst Neth. (2019) 131:134–8. doi: 10.1016/j.lungcan.2019.03.015 31027690

[5] Chamberlain M Junck L Brandsma D Soffietti R Rudà R Raizer J . Leptomeningeal metastases: a RANO proposal for response criteria. Neuro-Oncol. (2017) 19:484–92. doi: 10.1093/neuonc/now183 PMC5464328 28039364

[6] How J Mann J Laczniak AN Baggstrom MQ . Pulsatile erlotinib in EGFR-positive non-small-cell lung cancer patients with leptomeningeal and brain metastases: review of the literature. Clin Lung Cancer. (2017) 18:354–63. doi: 10.1016/j.cllc.2017.01.013 28245967

[7] Morris PG Reiner AS Szenberg OR Clarke JL Panageas KS Perez HR . Leptomeningeal metastasis from non-small cell lung cancer: survival and the impact of whole brain radiotherapy. J Thorac Oncol Off Publ Int Assoc Study Lung Cancer. (2012) 7:382–5. doi: 10.1097/JTO.0b013e3182398e4f 22089116

[8] Li Y-S Jiang B-Y Yang J-J Tu H-Y Zhou Q Guo W-B . Leptomeningeal metastases in patients with NSCLC with EGFR mutations. J Thorac Oncol Off Publ Int Assoc Study Lung Cancer. (2016) 11:1962–9. doi: 10.1016/j.jtho.2016.06.029 27539328

[9] Nosaki K Yamanaka T Hamada A Shiraishi Y Harada T Himeji D . Erlotinib for non-small cell lung cancer with leptomeningeal metastases: A phase II study (LOGIK1101). Oncol. (2020) 25:e1869–78. doi: 10.1634/theoncologist.2020-0640 PMC8108053 32654250

[10] Nanjo S Hata A Okuda C Kaji R Okada H Tamura D . Standard-dose osimertinib for refractory leptomeningeal metastases in T790M-positive EGFR-mutant non-small cell lung cancer. Br J Cancer. (2018) 118:32–7. doi: 10.1038/bjc.2017.394 PMC5765232 29190637

[11] Brastianos PK Carter SL Santagata S Cahill DP Taylor-Weiner A Jones RT . Genomic characterization of brain metastases reveals branched evolution and potential therapeutic targets. Cancer Discovery. (2015) 5:1164–77. doi: 10.1158/2159-8290.CD-15-0369 PMC4916970 26410082

[12] Ozcan G Singh M Vredenburgh Jj . Leptomeningeal metastasis from non-small cell lung cancer and current landscape of treatments. Clin Cancer Res Off J Am Assoc Cancer Res. (2023) 29:11–29. doi: 10.1158/1078-0432.CCR-22-1585 35972437

[13] Wang Y Yang X Li Nj Xu Jx . Leptomeningeal metastases in non-small cell lung cancer: Diagnosis and treatment. Lung Cancer Amst Neth. (2022) 174:1–11. doi: 10.1016/j.lungcan.2022.09.013 36206679

[14] Wang Y Jiang F Zhang Y Li M Li Y Li H . Unique genomic alterations of cerebrospinal fluid cell-free DNA are critical for targeted therapy of non-small cell lung cancer with leptomeningeal metastasis. Front Oncol. (2021) 11:701171. doi: 10.3389/fonc.2021.701171 34671549 PMC8522975

[15] Frankel D Nanni-Metellus I Robaglia-Schlupp A Tomasini P Guinde J Barlesi F . Detection of EGFR, KRAS and BRAF mutations in metastatic cells from cerebrospinal fluid. Clin Chem Lab Med. (2018) 56:851–6. doi: 10.1515/cclm-2017-0527 29306909

[16] Case report: Vemurafenib treatment in brain metastases of BRAFS365L -mutant lung papillary cancer by genetic sequencing of cerebrospinal fluid circulating tumor DNA detection (Accessed October 28, 2024). 10.3389/fonc.2021.688200 PMC8226071 34178685

[17] Chamberlain MC . Neoplastic meningitis. Oncol. (2008) 13:967–77. doi: 10.1634/theoncologist.2008-0138 18776058

[18] Patil V Noronha V Vallathol DH Menon N Mahajan A Janu A . Leptomeningeal metastasis from non-small cell lung cancer- a *post-hoc* analysis from four randomised clinical trials. Ecancermedicalscience. (2022) 16:1414. doi: 10.3332/ecancer.2022.1414 36072229 PMC9377816

[19] Freilich RJ Krol G DeAngelis LM . Neuroimaging and cerebrospinal fluid cytology in the diagnosis of leptomeningeal metastasis. Ann Neurol. (1995) 38:51–7. doi: 10.1002/ana.410380111 7611725

[20] Remon J Le Rhun E Besse B . Leptomeningeal carcinomatosis in non-small cell lung cancer patients: A continuing challenge in the personalized treatment era. Cancer Treat Rev. (2017) 53:128–37. doi: 10.1016/j.ctrv.2016.12.006 28110254

[21] Grossman SA Krabak MJ . Leptomeningeal carcinomatosis. Cancer Treat Rev. (1999) 25:103–19. doi: 10.1053/ctrv.1999.0119 10395835

[22] Le Rhun E Taillibert S Chamberlain MC . Carcinomatous meningitis: Leptomeningeal metastases in solid tumors. Surg Neurol Int. (2013) 4:S265–288. doi: 10.4103/2152-7806.111304 PMC3656567 23717798

[23] Wall M Falardeau J Fletcher WA Granadier RJ Lam BL Longmuir RA . Risk factors for poor visual outcome in patients with idiopathic intracranial hypertension. Neurology. (2015) 85:799–805. doi: 10.1212/WNL.0000000000001896 26245929 PMC4553022

[24] Mayer RR Frankfort BJ Strickland BA Debnam JM McCutcheon IE Groves MD . Leptomeningeal metastases presenting exclusively with ocular disturbance in 34 patients: A tertiary care cancer hospital experience. J Clin Neurosci Off J Neurosurg Soc Australas. (2017) 39:151–4. doi: 10.1016/j.jocn.2017.01.024 28215459

[25] Hida T Nokihara H Kondo M Kim YH Azuma K Seto T . Alectinib versus crizotinib in patients with ALK-positive non-small-cell lung cancer (J-ALEX): an open-label, randomised phase 3 trial. Lancet Lond Engl. (2017) 390:29–39. doi: 10.1016/S0140-6736(17)30565-2 28501140

[26] Dudnik E Siegal T Zach L Allen AM Flex D Yust-Katz S . Durable brain response with pulse-dose crizotinib and ceritinib in ALK-positive non-small cell lung cancer compared with brain radiotherapy. J Clin Neurosci Off J Neurosurg Soc Australas. (2016) 26:46–9. doi: 10.1016/j.jocn.2015.05.068 26677785

[27] Liao B-C Lee J-H Lin C-C Chen Y-F Chang C-H Ho C-C . Epidermal growth factor receptor tyrosine kinase inhibitors for non-small-cell lung cancer patients with leptomeningeal carcinomatosis. J Thorac Oncol Off Publ Int Assoc Study Lung Cancer. (2015) 10:1754–61. doi: 10.1097/JTO.0000000000000669 26334749

[28] Park JH Kim YJ Lee J-O Lee K-W Kim JH Bang S-M . Clinical outcomes of leptomeningeal metastasis in patients with non-small cell lung cancer in the modern chemotherapy era. Lung Cancer Amst Neth. (2012) 76:387–92. doi: 10.1016/j.lungcan.2011.11.022 22186628

[29] Naidoo J Schreck KC Fu W Hu C Carvajal-Gonzalez A Connolly RM . Pembrolizumab for patients with leptomeningeal metastasis from solid tumors: efficacy, safety, and cerebrospinal fluid biomarkers. J Immunother Cancer. (2021) 9:e002473. doi: 10.1136/jitc-2021-002473 34380662 PMC8359453

[30] Zheng M-M Tu H-Y Yang J-J Zhang X-C Zhou Q Xu C-R . Clinical outcomes of non-small cell lung cancer patients with leptomeningeal metastases after immune checkpoint inhibitor treatments. Eur J Cancer Oxf Engl 1990. (2021) 150:23–30. doi: 10.1016/j.ejca.2021.03.037 33882375

[31] Vallathol D Patil V Noronha V Joshi A Menon N Prabhash K . Leptomeningeal metastasis from extracranial solid tumors. Cancer Res Stat Treat. (2020) 3:254. doi: 10.4103/CRST.CRST_38_20

[32] Fan C Zhao Q Li L Shen W Du Y Teng C . Efficacy and safety of intrathecal pemetrexed combined with dexamethasone for treating tyrosine kinase inhibitor-failed leptomeningeal metastases from EGFR-mutant NSCLC-a prospective, open-label, single-arm phase 1/2 clinical trial (Unique identifier: chiCTR1800016615). J Thorac Oncol Off Publ Int Assoc Study Lung Cancer. (2021) 16:1359–68. doi: 10.1016/j.jtho.2021.04.018 33989780

[33] Cardenal F López-Cabrerizo MP Antón A Alberola V Massuti B Carrato A . Randomized phase III study of gemcitabine-cisplatin versus etoposide-cisplatin in the treatment of locally advanced or metastatic non–small-cell lung cancer. J Clin Oncol. (1999) 17:12–2. doi: 10.1200/JCO.1999.17.1.12 10458212

[34] Lee SJ Lee J-I Nam D-H Ahn YC Han JH Sun J-M . Leptomeningeal carcinomatosis in non-small-cell lung cancer patients: impact on survival and correlated prognostic factors. J Thorac Oncol Off Publ Int Assoc Study Lung Cancer. (2013) 8:185–91. doi: 10.1097/JTO.0b013e3182773f21 23328548

[35] Zorofchian S Iqbal F Rao M Aung PP Esquenazi Y Ballester LY . Circulating tumour DNA, microRNA and metabolites in cerebrospinal fluid as biomarkers for central nervous system Malignancies. J Clin Pathol. (2019) 72:271–80. doi: 10.1136/jclinpath-2018-205414 30467241

[36] Li Y-S Zheng M-M Jiang B-Y Tu H-Y Yang J-J Zhang X-C . Association of cerebrospinal fluid tumor DNA genotyping with survival among patients with lung adenocarcinoma and central nervous system metastases. JAMA Netw Open. (2020) 3:e209077. doi: 10.1001/jamanetworkopen.2020.9077 32749467 PMC7403922

[37] Zheng M-M Li Y-S Jiang B-Y Tu H-Y Tang W-F Yang J-J . Clinical utility of cerebrospinal fluid cell-free DNA as liquid biopsy for leptomeningeal metastases in ALK-rearranged NSCLC. J Thorac Oncol Off Publ Int Assoc Study Lung Cancer. (2019) 14:924–32. doi: 10.1016/j.jtho.2019.01.007 30659989

[38] Li N Liu Y Duan J Yang B Bai H Sun R . Prognostic significance of molecular characteristics of cerebrospinal fluid for non-small cell lung cancer patients with leptomeningeal metastasis. Thorac Cancer. (2019) 10:1673–82. doi: 10.1111/1759-7714.13123 PMC6669802 31368671

[39] Cardarella S Ogino A Nishino M Butaney M Shen J Lydon C . Clinical, pathologic, and biologic features associated with BRAF mutations in non-small cell lung cancer. Clin Cancer Res Off J Am Assoc Cancer Res. (2013) 19:4532–40. doi: 10.1158/1078-0432.CCR-13-0657 PMC3762878 23833300

[40] Paik PK Arcila ME Fara M Sima CS Miller VA Kris MG . Clinical characteristics of patients with lung adenocarcinomas harboring BRAF mutations. J Clin Oncol Off J Am Soc Clin Oncol. (2011) 29:2046–51. doi: 10.1200/JCO.2010.33.1280 PMC3107760 21483012

[41] Planchard D Besse B Groen HJM Souquet P-J Quoix E Baik CS . Dabrafenib plus trametinib in patients with previously treated BRAF(V600E)-mutant metastatic non-small cell lung cancer: an open-label, multicentre phase 2 trial. Lancet Oncol. (2016) 17:984–93. doi: 10.1016/S1470-2045(16)30146-2 PMC4993103 27283860

[42] Tissot C Couraud S Tanguy R Bringuier P-P Girard N Souquet P-J . Clinical characteristics and outcome of patients with lung cancer harboring BRAF mutations. Lung Cancer Amst Neth. (2016) 91:23–8. doi: 10.1016/j.lungcan.2015.11.006 26711930

[43] Johnson DB Zhao F Noel M Riely GJ Mitchell EP Wright JJ . Trametinib activity in patients with solid tumors and lymphomas harboring BRAF non-V600 mutations or fusions: results from NCI-MATCH (EAY131). Clin Cancer Res Off J Am Assoc Cancer Res. (2020) 26:1812–9. doi: 10.1158/1078-0432.CCR-19-3443 PMC7165046 31924734

[44] Miyauchi S Shien K Takeda T Araki K Nakata K Miura A . Antitumor effects of pan-RAF inhibitor LY3009120 against lung cancer cells harboring oncogenic BRAF mutation. Anticancer Res. (2020) 40:2667–73. doi: 10.21873/anticanres.14237 32366411

